# Longitudinal Differences in Human Hippocampal Connectivity During Episodic Memory Processing

**DOI:** 10.1093/texcom/tgaa010

**Published:** 2020-04-14

**Authors:** Kyuwan Choi, Lisa Bagen, Linley Robinson, Gray Umbach, Michael Rugg, Bradley Lega

**Affiliations:** 1 Department of Neurosurgery, University of Texas Southwestern, Dallas, TX 75390, USA; 2 Center for Vital Longevity, University of Texas at Dallas, Dallas, TX 75235, USA; 3 School of Behavioral and Brain Sciences, University of Texas at Dallas, Dallas, TX 75080, USA; 4 Department of Psychiatry, University of Texas Southwestern, Dallas, TX 75390, USA

**Keywords:** anterior hippocampus, episodic memory encoding, episodic memory retrieval, functional connectivity, posterior hippocampus

## Abstract

The question of longitudinal hippocampal functional specialization is critical to human episodic memory because an accurate understanding of this phenomenon would impact theories of mnemonic function and entail practical consequences for the clinical management of patients undergoing temporal lobe surgery. The implementation of the robotically assisted stereo electroencephalography technique for seizure mapping has provided our group with the opportunity to obtain recordings simultaneously from the anterior and posterior human hippocampus, allowing us to create an unparalleled data set of human subjects with simultaneous anterior and posterior hippocampal recordings along with several cortical regions. Using these data, we address several key questions governing functional hippocampal connectivity in human memory. First, we ask whether functional networks during episodic memory encoding and retrieval are significantly different for the anterior versus posterior hippocampus (PH). We also examine how connections differ across the 2–5 Hz versus 4–9 Hz theta frequency ranges, directly addressing the relative contribution of each of these separate bands in hippocampal–cortical interactions. While we report some overlapping connections, we observe evidence of distinct anterior versus posterior hippocampal networks during memory encoding related to frontal and parietal connectivity as well as hemispheric differences in aggregate connectivity. We frame these findings in light of the proposed AT/PM memory systems. We also observe distinct encoding versus retrieval connectivity patterns between anterior and posterior hippocampal networks, we find that overall connectivity is greater for the PH in the right hemisphere, and further that these networks significantly differ in terms of frontal and parietal connectivity. We place these findings in the context of existing theoretical treatments of human memory systems, especially the proposed AT/PM system. During memory retrieval, we observe significant differences between slow-theta (2–5 Hz) and fast-theta (4–9 Hz) connectivity between the cortex and hippocampus. Taken together, our findings describe mnemonically relevant functional connectivity differences along the longitudinal axis of the human hippocampus that will inform interpretation of models of hippocampal function that seek to integrate rodent and human data.

## Introduction

The question of whether the anterior and posterior hippocampus (PH) serve different or complementary functional roles has been a subject of debate in the cognitive neuroscience of human memory for over a generation (Zeidman and Maguire 2016; Copara et al. 2014; Strange et al. 2014; Grady 2019). While this question has mainly been addressed in humans with fMRI, a much wider range of methods has been employed with experimental rodents, including single unit and local field potential recordings. A principal reason that this issue has not been studied extensively using electrophysiological methods in humans is that, historically, human studies have exclusively employed anterior hippocampal electrodes that limit the ability to make directional anterior versus posterior connectivity comparisons. This entails that existing functional connectivity studies utilize anterior hippocampal contacts (Burke et al. 2013; Solomon et al. 2017; Fell et al. 2002), whereas most memory-related findings in rodents have come from the dorsal hippocampus; more analogous to the posterior area in humans (Zemla and Basu 2017; Broadbent et al. 2004; Clark et al. 2000; Knierim 2015). A number of theories have been offered to describe the longitudinal differences in hippocampal function (Lepage et al. 1998; Ryan et al. 2010; Fanselow and Dong 2010; Kerr et al. 2007). Human functional imaging studies suggest that anterior and posterior regions are preferentially connected with different cortical and subcortical areas. Some of these functional connectivity differences, such as stronger posterior hippocampal connections with the parahippocampal and cingulate cortex, and stronger anterior connections with the temporal pole, map onto a proposed distinction between anterior and posterior cortical “memory systems” (Bubb et al. 2017; Bonner and Price 2013; Zeidman and Maguire 2016), although other observations (such as stronger posterior–dorsolateral prefrontal cortex [DLPFC] connectivity [Poppenk and Moscovitch 2011]) do not. However, the majority of existing functional connectivity data have come from resting state fMRI (Ankudowich et al. 2019; Avery et al. 2018; Wang et al. 2006), and to our knowledge there has been no direct comparison of anterior versus posterior hippocampal connectivity using direct brain recordings during the encoding and retrieval of episodic memories. As such, further characterization of human hippocampal functional connectivity that takes account of longitudinal functional differences is critical to advance the understanding of human memory. Here, we use phase synchronization to separately examine anterior versus posterior functional hippocampal connections as human intracranial electroencephalography (EEG) patients perform an episodic memory task.

A parallel question regarding human hippocampal neurophysiology pertains to theta oscillations. In rodents, a single 3–9 Hz oscillation is observed both in the hippocampus and neocortex which exhibits numerous functionally significant properties during memory encoding, including power increases that predict successful encoding, phase amplitude coupling, and connectivity between different medial temporal structures as well as with cortical regions such as the medial frontal cortex (Deadwyler et al. 2013; Kim et al. 2013) (although see [Jacobs 2014] regarding the existence of two separate theta bands in rodents). In humans, this is not the case: studies have consistently reported memory-relevant oscillatory activity in at least two distinct bands, comprising a slower oscillation between 2 and 5 Hz and higher frequency oscillations between 4 and 9 Hz. The latter oscillations seem to predominate in cortical locations, often as part of low frequency desynchronization and associated decrease in oscillatory power related to successful memory formation (Watrous et al. 2013; Bush et al. 2017; Jacobs 2014). By contrast, in the hippocampus, power increases predicting memory encoding success and phase amplitude coupling occur preferentially in the 2–5 Hz range in both episodic memory and spatial navigation studies (Jacobs et al. 2017; Lin et al. 2017). A natural question to ask therefore is whether one or more cortical regions preferentially interact with the hippocampus in the slower (2–5 Hz) or faster (4–9 Hz) frequency ranges. To our knowledge, a focused analysis addressing this question has not been performed in humans, and nor has the question of preferential anterior versus posterior connectivity (as discussed above) been linked to the issue of separate theta frequency ranges relevant for successful memory formation. Nonetheless, existing studies of human functional connectivity during mnemonic processing principally describe data obtained from hippocampal depth electrodes inserted via open craniotomy, and mainly target the anterior portion of the hippocampus (Staresina et al. 2012; Staresina et al. 2013). Connectivity analyses conducted using these data sets have generally aggregated hippocampal signals across different recording locations, and collapsed oscillations from 2 to 10 Hz into a single broad frequency band. Here, we investigated hippocampal connectivity using a novel data set including electrodes in both the anterior and PH.

Using intracranial depth electrodes, here we investigated the connectivity of the anterior and PH with 8 memory-relevant cortical regions (Frontal lobe: DLPFC, ventrolateral prefrontal cortex [VLPFC]; medial temporal lobe: parahippocampal cortex [PHC], entorhinal cortex [ERC], posterior lateral temporal cortex [PLT]; Parietal lobe: lateral parietal cortex [LPC] [representing a combination of supramarginal and angulary gyrus], anterior lateral temporal cortex [ALT]; posterior cingulate cortex [PCC]) across three frequency bands during memory encoding and retrieval. Following prior studies, we quantified connectivity in the two theta bands as discussed above, as well as in the low gamma frequency range to provide a comprehensive examination of hippocampal functional connectivity. To presage our results, we observed significant connectivity differences that have implications for models of mnemonic processing and hippocampal longitudinal specialization as well as functional distinctions within the theta frequency band. In terms of memory models, our results identify distinct anterior and posterior hippocampal networks, especially during memory encoding, as well as overall hemispheric differences in connection strength. Unexpectedly, the posterior network included frontal lobe locations, while the anterior network included the posterior cingulate as well as parietal cortex and PHC during encoding, and we discuss how these findings inform the AT/PM model described above. Further, we identify distinct encoding versus retrieval hippocampal networks and theta–frequency connectivity differences that impact synchronization models described in earlier iEEG analyses (Solomon et al. 2017). Finally, our findings inform theories of anterior/posterior functional differences in episodic memory related to encoding/retrieval differences and spatial versus nonspatial processing, along with highlighting the importance of hemispheric considerations in these models.

## Materials and Methods

### Participants

A total of 91 patients with medically intractable epilepsy who underwent stereoelectroencephalography surgery for clinical purposes were recruited to participate in this study. Participants came from the UT Southwestern epilepsy surgery program across a time span of 4 years. Participants had intracranial depth electrodes implanted at locations specified on the basis of clinical need by the neurology team, and electrodes were laterally inserted into the specified regions with robotic assistance. Among the 91 patients, 76 patients have bihemispheric coverage and interhemispheric connectivity was not measured. The demographic information of participants is summarized in Table 1. The research protocol was approved by the UT Southwestern Medical Center Institutional Review Board, and each participant gave informed consent prior to data collection. Following implantation, electrode localization was achieved by coregistration of the postoperative computer tomography scans with preoperative magnetic resonance images using the FLIRT software package (https://fsl.fmrib.ox.ac.uk/fsl/fslwiki/FLIRT). The coregistered images were evaluated by a member of the neuroradiology team to determine the final electrode locations. Electrodes from hemisphere of evident radiographic abnormalities including temporal sclerosis or previous neurosurgery were excluded from analysis. We aggregated electrodes into a set of 10 regions: anterior hippocampus (AH), PH, DLPFC, VLPFC, PLT, PCC, PHC, LPC, ALT, and ERC. All regions had at least 11 subjects contributing data to each region.

**Table 1:** Demographic information of participants

Participant	Age	Sex	Duration of epilepsy (years)	Temporal lobe seizure onset
Participant 1	59	F	48	Yes
Participant 2	21	M	5	No
Participant 3	40	F	10	No
Participant 4	47	F	10	No
Participant 5	25	M	13	Yes
Participant 6	36	F	34	Yes
Participant 7	47	M	5	No
Participant 8	44	F	4	Yes
Participant 9	64	M	64	Yes
Participant 10	38	M	37	Yes
Participant 11	45	F	38	No
Participant 12	44	M	4	No
Participant 13	24	M	18	Yes
Participant 14	29	M	3	Yes
Participant 15	37	M	33	No
Participant 16	25	M	13	No
Participant 17	36	F	2	No
Participant 18	25	F	22	No
Participant 19	32	M	20	No
Participant 20	63	M	4	No
Participant 21	46	F	11	No
Participant 22	32	F	25	No
Participant 23	44	F	41	No
Participant 24	29	M	3	Yes
Participant 25	24	M	6	No
Participant 26	43	M	20	No
Participant 27	40	F	5	No
Participant 28	21	M	9	Yes
Participant 29	32	F	7	No
Participant 30	22	M	3	No
Participant 31	40	M	7	No
Participant 32	20	F	3	Yes
Participant 33	37	M	35	No
Participant 34	51	F	28	No
Participant 35	38	F	14	No
Participant 36	51	F	43	No
Participant 37	22	M	8	No
Participant 38	32	M	28	No
Participant 39	37	F	15	Yes
Participant 40	43	F	19	Yes
Participant 41	36	M	29	No
Participant 42	34	M	24	No
Participant 43	33	F	4	No
Participant 44	62	M	17	No
Participant 45	31	F	6	No
Participant 46	60	M	9	No
Participant 47	38	M	34	No
Participant 48	25	F	11	No
Participant 49	55	F	41	No
Participant 50	35	M	27	Yes
Participant 51	29	M	10	Yes
Participant 52	50	M	42	No
Participant 53	27	F	12	No
Participant 54	29	M	14	No
Participant 55	46	F	33	Yes
Participant 56	55	M	30	No
Participant 57	36	F	19	No
Participant 58	20	M	1	No

### Free-Recall Task

Each subject participated in a verbal free-recall task in which they studied a list of words under instructions to commit each item to memory. The task was performed at bedside on a laptop. Analog pulses were used to synchronize experimental events with the recorded iEEG signals. The task comprised three phases: encoding, delay, and retrieval. In the encoding phase, lists of 12 words were visually presented. Words were selected at random from a pool of high frequency English nouns (http://memory.psych.upenn.edu/WordPools). The same word pool was used for each subject, but for each subject a new set of lists was generated for all sessions (to avoid repetition of an item across sessions). To calculate word frequency scores, we counted the number of occurrences of all words in the word pool across 500 000 Wikipedia documents obtained from a public source (Shaoul et al. 2010). We then divided the number of occurrences across all documents, by the number of documents scanned to obtain a frequency value for each word. The minimum value of frequency is 3.8 ×${10}^5$and the maximum value is 0.61 occurrences/document. To calculate semantic similarity scores between all words in our word pool, we performed latent semantic analysis. First, we generated a word count matrix by tracking all occurrences of each word in the word pool across 100 000 documents from the aforementioned text corpus. We then performed singular value decomposition, yielding the following data structures:

*W* = *U* × *S* × *V^T^*where *W* is the word count matrix, *U* contains the left singular vectors, *S* contains the singular values for each semantic dimension along its diagonal, and *V* contains the right singular vectors. Matrix multiplication of *U* and *S* yields a word-by-semantic dimension matrix, with each row corresponding to a word. Calculating the cosine similarity between rows of this matrix yields the semantic similarity between words.

The 99th percentile of semantic similarity scores was 0.0863. Each word was presented for 1600 ms, followed by a blank interstimulus interval that varied between 750 and 1000 ms. We tested for the impact of the jitter used for items, comparing the interstimulus interval for recalled versus nonrecalled items. This revealed no difference (*t*(188) < 0.01, *P* = 0.99, Cohen’s *d* = 0.003). A total of 706 unique words were presented across participants, with a mean length of 4.97 ± 1.57 letters. Prior to each word list, subjects were given a 10-s countdown period in which they passively watched the monitor as centrally presented digits counted down from 10. At the conclusion of each study list, a postencoding delay followed for 20 s. During the delay, participants performed an arithmetic task (A + B = C?) to limit rehearsal. Following the delay period, the recall period was signaled by a 300 ms 60 Hz tone. Participants were instructed to verbally recall as many words as possible from the word list within a 30 s recall period (memory retrieval). This was done 25 times per session and participants completed between one and three sessions. Verbal responses were digitally recorded and parsed offline using Penn TotalRecall (http://memory.psych.upenn.edu/TotalRecall). Further details of the task are described in Sederburg et al. (2003).

### Electrocorticographic Recordings

iEEG signals were recorded using depth electrodes (contacts spaced 5–10 mm apart) using a Nihon-Kohden EEG-2100 clinical system. Signals were sampled at 1000 Hz and referenced to a common intracranial contact. Raw signals were subsequently rereferenced to a bipolar montage, with each contact referenced to the superficial adjacent contact (Solomon et al. 2017). Bipolar signals were notch filtered at 60 Hz with a fourth order 2 Hz stopband butterworth notch filter in order to remove the effects of line noise on the iEEG signals (Solomon et al. 2017). We excluded activity from electrodes that were located at the site of seizure onset locations or frequent interictal activity. Interictal artifact was eliminated using a kurtosis algorithm as previously published (Lin et al. 2017). Channels exhibiting highly nonphysiologic signal due to damage or misplacement were excluded prior to rereferencing based upon review of clinical information regarding seizure onset and interictal activity. Table 2 shows the number of subjects with electrodes located in each region of interest.

**Table 2:** Number of subjects with electrodes localized to each region

Regions/hemisphere	Left	Right
AH	43	35
PH	30	29
DLPFC	20	17
VLPFC	22	11
PLT	27	27
PCC	32	24
PHC	16	18
LPC	26	18
ERC	25	17
ALT	50	46

### Phase Analysis

In order to test for the presence of two distinct theta bands in the anterior and PH, we used the multiple oscillations detection algorithm (MODAL). However, we selected the MODAL because it adaptively identifies oscillatory band(s) without introducing experimenter bias regarding bands of interest, and that it excludes periods when phase is noisy because oscillations are absent. We implemented the algorithm as has been previously described (Watrous et al. 2018). Alternative methods, especially the fitting oscillations and one-over-f algorithm, have also been developed although we have less experience with their application (Haller et al. 2018). This analysis ensured that the distinct rhythms detected by MODAL did not arise from distinct populations of electrodes with Yule’s Q. In each 1800 ms time window beginning at the appearance of a given study item in the encoding list, the oscillatory phase was extracted at each time–frequency pixel using Morlet wavelets with a width of six, at 49 log-spaced frequencies centered at 2^(*n*/8)^, *n* = 8:64 (Tallon-Baudry et al. 1997). In these time windows, the phase locking values (PLV) between the hippocampus and various cortical regions were calculated for successful and unsuccessful trials for each electrode pair. ∆PLV_encoding_ was obtained by subtracting the calculated PLV_unsuccess_ (PLV from unsuccessful trials) from the PLV_success_ (PLV from successful trials) as shown in eq. 1. A phase locking statistic (PLS) (Lachaux et al. 1999) was then obtained by comparing the ∆PLV_encoding_ with 2000 values generated from shuffling successful and unsuccessful trial labels prior to calculating phase coherence. A normal inverse transformation was applied to the PLS values to convert them to *Z*-values. These were averaged across all electrode pairs in a given region—hippocampal connection to provide a single value per subject per region—hippocampal connection. These *Z*-value distributions were compared with a null hypothesis of zero (no functional connection) using a one-sample, two-sided *t*-test. A false discovery rate (FDR) of *q* < 0.2 (Benjamini and Hochberg 1995) was used to correct for multiple comparisons for all analyses, since *q*-values between 0.1 and 0.2 after FDR correction are known to be acceptable for this purpose (Genovse et al. 2002). Connections whose *Z*-score distribution significantly deviated from zero as determined by a one-sample, two-sided *t*-test rendering a *P*-value less than 0.05, and which passed FDR correction at a *q*-value of less than 0.2, were passed on to the null network test.

As a second method to identify significant connections and following previously published work (Solomon et al. 2017), we employed a shuffled distribution across all possible connections. In total, 500 null networks generated from shuffled trials were used to generate the distribution of chance network-level statistics. These null networks were constructed by shuffling trial labels (recalled and nonrecalled items in the case of encoding, retrieval and deliberation items in the case of retrieval) for every hippocampal connection in our data set (both hemispheres: anterior and posterior). The true connectivity values for each region (single value per frequency band, averaged across all electrode pairs) were compared with the null distributions in order to obtain a *P*-value. Only in the case where the *P*-value was less than 0.05 for this test and if it passed FDR for the within-region PLS calculation (described above) did we considered the connectivity of the region to be significant and include it in further analysis.

The connections that passed these two tests were defined as significant connections. To compare the mean value of the *Z*-score of anterior and PH, the absolute value of the mean of the *Z*-scores were aggregated and a two-sample paired *t*-test with FDR correction was applied. In order to compare the significant connections of anterior and PH in each frequency band of interest, the absolute values of the mean of z-score were divided into three frequency bands (slow theta (2–5 Hz), fast theta (5–9 Hz), and gamma bands (40–70 Hz)) and compared with a two-sample paired *t*-test with FDR correction. We elected to use absolute value to account for both significant synchronous and desynchronous connections and to limit the total number of comparisons made:

(1) \begin{equation*} \Delta \mathrm{PL}{\mathrm{V}}_{\mathrm{encoding}}=\mathrm{PL}{\mathrm{V}}_{\mathrm{success}}-\mathrm{PL}{\mathrm{V}}_{\mathrm{unsuccess}}. \end{equation*}

For the retrieval analysis, we defined the retrieval period as the 900 ms preceding onset of vocalization of the recalled word. We used 900 ms from the cross-fixation period as a baseline comparator, and the PLV was obtained from the retrieval and baseline period. ∆PLV_retrieval_ was obtained by subtracting the calculated PLV_baseline_ from the PLV_retrieval_ as shown eq. 2:

(2) \begin{equation*} {\Delta \mathrm{PLV}}_{\mathrm{retrieval}}={\mathrm{PLV}}_{\mathrm{retrieval}}\hbox{-} {\mathrm{PLV}}_{\mathrm{baseline}}. \end{equation*}

Then, the PLS was calculated as for the encoding period, and the resulting PLS values were converted to *Z*-values. The *Z*-values were then grouped by brain region and used for statistical analyses.

## Results

We sought to investigate hippocampal functional connectivity utilizing a data set that permitted us to directly compare anterior versus posterior hippocampal connections. All subjects contributing to the data set had both anterior and posterior hippocampal contacts, although not all subjects contributed data to each cortical region. We aggregated electrodes into a set of 10 regions, namely: AH, PH, DLPFC, VLPFC, lateral temporal cortex, posterior temporal cortex, parahippocampal gyrus, LPC, lateral middle temporal cortex, and ERC. The number of individuals contributing electrodes to each of the regions is detailed in Table 2. Our goals were to assess differential connectivity across the hippocampal axis, also testing whether different connections preferentially occur in the slow theta (2–5 Hz), fast theta (5–9 Hz), or gamma frequency ranges (40–70 Hz). Across participants, the average probability of recall for all words was 25.45% (SD = ±9.95%). The average percentage of list intrusions (recall errors) per subject was 5.02%. These were excluded from analysis. All patients recalled at least 10% of items (minimum criteria for inclusion of data). A regression model predicting memory performance using the predictors of age, sex, duration of epilepsy, and presence or absence of temporal lobe epilepsy did not identify any significant association between these variables and memory behavior (*P* > 0.50 for all predictors).

### Both Fast and Slow Theta Oscillations are Present in the Anterior and PH

**Figure 1:**
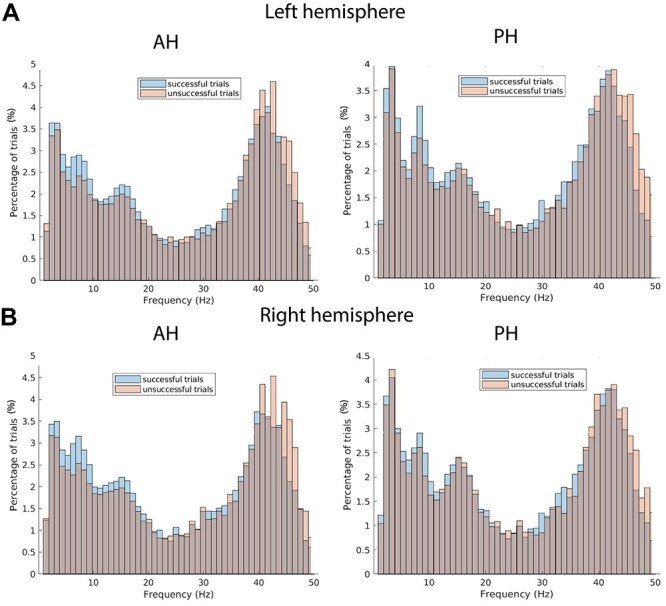
Hippocampal oscillations related to memory encoding. (*A*) Histograms showing the results of applying the peak detection algorithm to all trials in our data for successful and unsuccessful trials on anterior (AH) and posterior (PH) hippocampus in the left hemisphere (every trial in data set included). (*B*) The results for the right hemisphere.

For our first analysis, we asked whether slow and fast theta oscillations were present in the anterior and PH as determined by a previously validated oscillation detection algorithm (MODAL [Cohen 2014]). This analysis was governed by the hypothesis that both types of oscillations would be present in both the posterior and AH based upon existing data (Lega et al. 2011). The plots in Fig. 1 reflect the distribution of detected oscillations across our data set (shown as percent of trials exhibiting an oscillation at each frequency). We observed peaks in this plot centered at approximately 3 Hz (slow-theta range) and 8 Hz (fast-theta range), as well as in the low gamma band (45 Hz). We next asked whether the same electrodes exhibited both oscillations (oscillations present simultaneously in a given electrode), or if a different population of electrodes was contributing to oscillatory peaks within each theta frequency band. We tested for an interaction in the identity of slow-theta and fast-theta providing electrodes using Yule’s Q (Yule 1912). This revealed that the electrodes that exhibited slow-theta oscillatory peaks also tended to show fast theta oscillations (Yule’s Q = 0.689, *P* < 0.01), suggesting the peaks observed in the histograms reflect distinct oscillations occurring simultaneously in the same hippocampal region (not a separate population of slow versus fast theta individuals, or a single broad peak detected by MODAL). The result is consistent with previous findings (Lega et al. 2011).

### Distinct Anterior Versus Posterior Hippocampal Networks Support Memory Encoding

**Figure 2:**
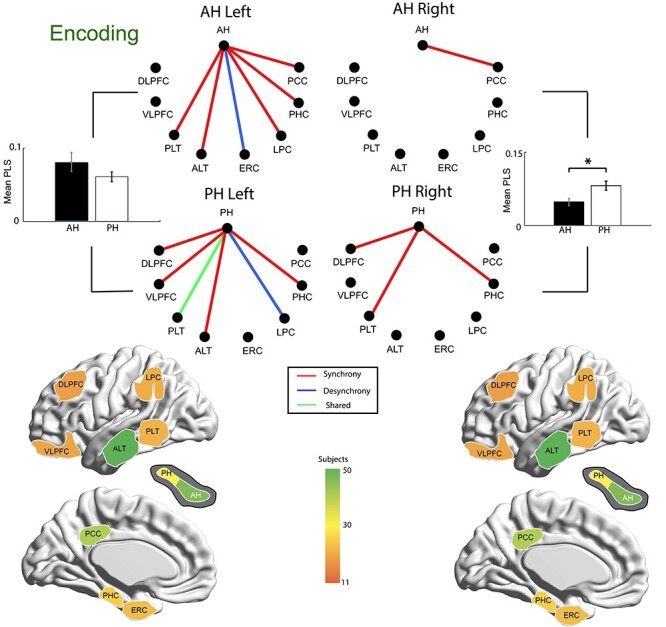
Functional connections of anterior and posterior hippocampus during item encoding. Significant functional connections are shown; each line reflects significant connectivity differences between successful and unsuccessful encoding across subjects, with every subject contributing both anterior and posterior hippocampal electrodes to each connection. Synchrony (represented in red) indicates greater connectivity for successful trials and desynchrony (represented in blue) shows decreased connectivity for successful trials, aggregated across 3 frequency bands (slow theta, fast theta, and gamma connections contributing to each line). Green lines indicate different synchronous/desynchronous connections by band. Bar graphs show mean connectivity (absolute value, to account for significant synchronous and desynchronous connections) across all cortical locations for anterior and posterior hippocampus. ^*^P < 0.05, paired *t*-test.

We began our connectivity analysis by using the PLS method (Lachaux et al. 1999) to calculate a functional connectivity weight between the anterior and PH and each of eight cortical regions. This utilized a null distribution generated by shuffling trial labels. This analysis was motivated by the hypothesis that different anterior versus posterior functional networks support memory encoding. Critically, for each cortical region of interest, subjects contributed both an anterior and posterior hippocampal electrode, reducing the possibility of bias due to different patient populations contributing either anterior or posterior electrode contacts. These PLS values across all electrode pairs in a given hippocampal-cortical connection were then tested against a null hypothesis of no successful/unsuccessful difference across subjects using a one sample *t*-test (against zero), with significant connections defined as those with an FDR corrected *P*-value (across all hippocampal region connections) of *P* < 0.05. We added an additional shuffle step, using “null networks” generated by shuffling trial labels and calculating connectivity for all possible connections. This was done separately for each of three frequency bands, and the results are shown in Fig. 2. The connections shown passed both the initial (within region) and network wide significance tests. We plotted those connections demonstrating increased connectivity (synchronous connections) in red and decreased connectivity (desynchronous connections) in blue. We quantified the aggregate functional connectivity for anterior and PH using the absolute value of the PLS statistic (to account for both synchronous and desynchronous connections) to compare anterior versus posterior hippocampal connectivity strength (one mean PLS value per hippocampal-cortical connection for each of the three frequency bands). We observed a significant difference in the right hemisphere, with stronger posterior hippocampal connectivity overall (paired *t*-test across all 24 possible connections, *t*(23) = 3.82, corrected *P* < 0.05, Cohen’s *d* = 1.19) and an interaction between anterior/PH and hemisphere (*F*(1,94) = 14.59, p < 0.05, η^2^ = 0.13).

We next directly compared each cortical connection between the anterior and PH in a pairwise matter, taking advantage of the presence of both anterior and posterior electrode contacts for each subject in our data set. As above, we hypothesized that memory encoding relies on different anterior versus posterior functional networks, and specifically we expected to observe greater anterior connectivity with prefrontal/anterior temporal areas but greater posterior connectivity with parietal regions. This was based in part on the proposed AT/PM system, examined in more detail in the Discussion section below. We directly tested a given region—anterior versus posterior connection only if at least one of these cortical-hippocampal connections (anterior or posterior) was significant in our initial network analysis (that is exhibited significant connectivity difference for successful versus unsuccessful encoding) such that only significant functional connections were tested for an a/p difference. Fig. 4 shows individual connections that were significantly different (FDR corrected *P*-value < 0.05, with paired *t*-test across connections of all subjects contributing to a given hippocampal-cortical connection), separately for synchronous versus desynchronous ones. An unexpected finding was significant synchronization between the AH and parietal brain regions (lateral parietal and posterior cingulate). Posterior hippocampal-lateral parietal connectivity exhibited desynchrony, whereas frontal connectivity was relatively stronger for posterior than for the anterior hippocampal connections. We followed up on this observation by directly quantifying connectivity for these regions, separated by hemisphere, by taking the average PLS value across the three frequency bands. These results are shown in Fig. 4, revealing that there is a significant interaction between A/P connectivity and frontal/parietal brain location (*F*(1,47) = 5.65, *P* < 0.05, η^2^ = 0.11) in the left hemisphere. In the aggregate data, this effect was driven by greater posterior hippocampal connectivity with frontal cortical locations (for frontal cortex: paired *t*-test, *t*(14) = 6.06, FDR corrected *P* < 0.05, Cohen’s *d* = 2.56, and for parietal cortex: paired *t*-test, *t*(14) = 2.69, FDR corrected *P* < 0.05, Cohen’s *d* = 1.59). In the right hemisphere, there was no significant interaction *F*(1,47) = 1.76, *P* > 0.05, η^2^ = 0.03).

### Slow and Fast Theta Networks are Similar During Item Encoding

**Figure 3:**
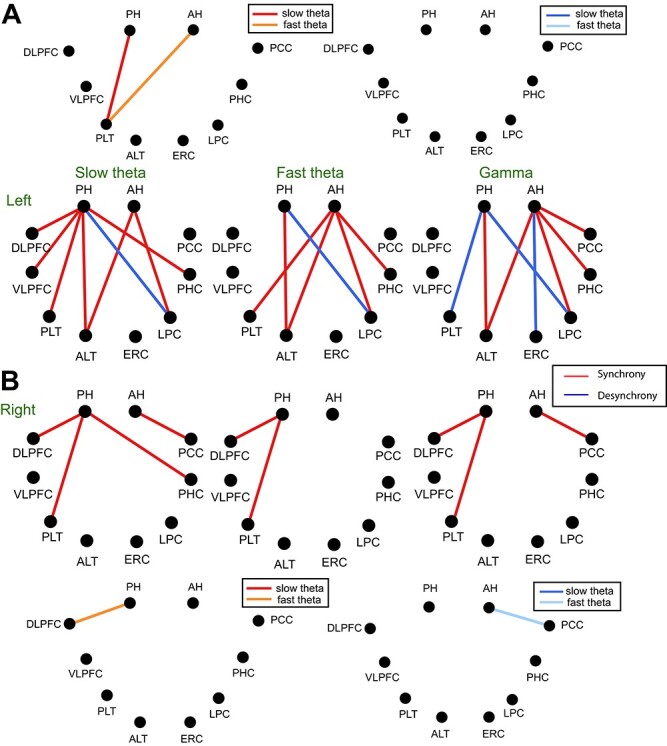
Functional connections of slow theta, fast theta, and gamma bands during item encoding for anterior and posterior hippocampus. (*A*) Results for left hemisphere. Upper figure shows the comparison between slow and fast theta band. The connectivity of slow theta is compared with that of fast theta in each region during encoding. Synchronous connectivity is shown in hot colors (red, orange), and desynchrous connectivity in cold colors (blue, light blue). Only the connections are shown when *P*-value is less than 0.05 with paired t test. In synchrous connectivity, slow theta is represented with red, and fast theta shown with orange. In desynchrous connectivity, slow theta is shown with blue, and fast theta represented with light blue. In the lower figure, red color indicates greater increased connectivity for successful encoding and blue color shows decreased connectivity. When breaking down connections by band, there are no significant differences between anterior and posterior, although all three bands exhibit stronger posterior than anterior connectivity in the right hemisphere, consistent with the aggregate results shown above. (*B*) Results for right hemisphere.

We hypothesized that posterior hippocampal connections would be more prevalent in the slow theta frequency band based upon observations of power differences during memory encoding (Lin et al. 2017). Overall however there was not a significant effect of frequency band on connectivity strength (*F*(2,95) = 0.21, *P* > 0.05, η^2^ = 0.0046) or an interaction with A/P location (*F*(2,95) = 0.01, *P* > 0.05, η^2^ = 0.0003). We also directly compared individual connections in a pairwise manner to identify those that are significantly different between slow versus fast-theta oscillations, these are shown in Fig. 3. This indicates that while aggregate connectivity was similar across both bands, specific connections preferentially occur in one or the other theta frequency range. One noteworthy finding is that hippocampal connectivity with the PLT occurs in the fast-theta range for the AH but in the slow-theta range for the PH in the left hemisphere. Additional differences in theta connectivity for specific connections are shown in Fig. 3. Theta frequency connectivity differences were more pronounced during item retrieval, discussed below.

**Figure 4:**
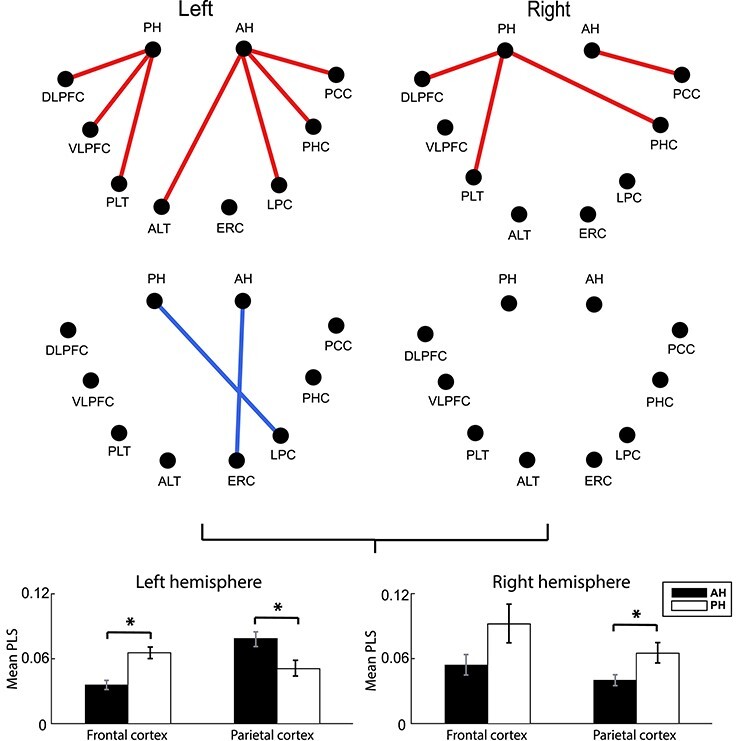
Difference in functional connections of anterior versus posterior hippocampus during item encoding. Red color represents a significantly different synchronous connection and blue color a significantly different desynchronous one. All significant connections (as shown in Fig. 2) were tested across all subjects contributing data to each connection. The bar plots below show the mean value of anterior and posterior hippocampal connections with the frontal (dorsolateral prefrontal cortex and VLPFC) and parietal cortex (lateral parietal cortex and posterior cingulate cortex). There is a significant difference between anterior and posterior hippocampus both for frontal and parietal cortex in the left hemisphere, and in the right hemisphere the parietal cortex difference is significant but with the opposite pattern (paired *t*-test, FDR corrected *P* < 0.05).

**Figure 5:**
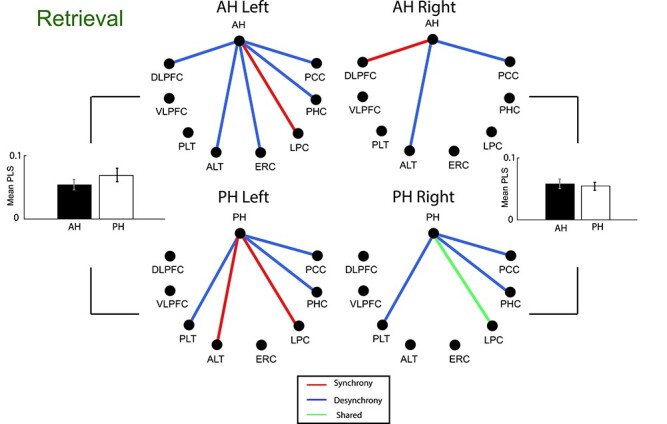
Anterior and posterior hippocampal functional connectivity during item retrieval. Hippocampal-cortical connections that exhibited significant connectivity differences between active item retrieval and baseline (deliberation period) are shown, with red indicating synchronous and blue desynchronous connections. The connections for which one band was synchronous and another desynchronous are shown in blue (see Fig. 6 for breakdown by band). The mean z-score of all anterior and posterior connections (absolute value) are represented by a bar graph for left and right hemisphere (^*^ mark represents *P* < 0.05, two-tailed paired *t*-test). There is no difference between anterior and posterior hippocampus both for left and right hemisphere (for left hemisphere, paired *t*-test, *P* > 0.05, for right hemisphere, paired *t*-test, *P* > 0.05).

**Figure 6:**
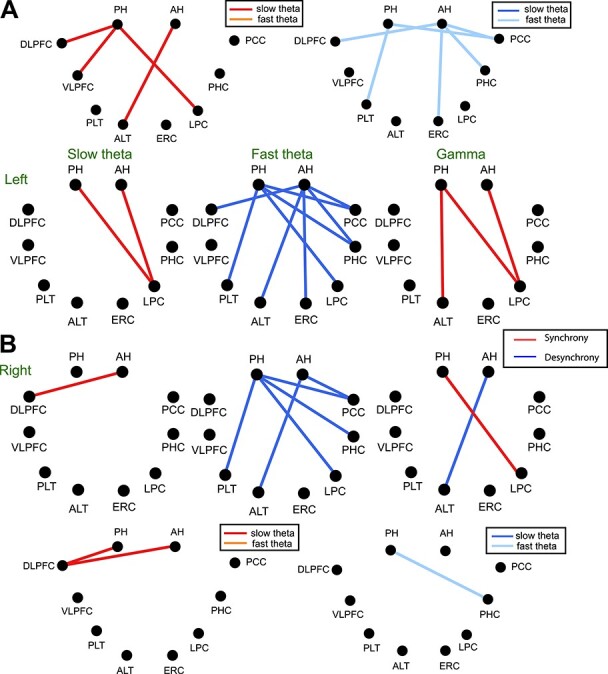
Functional connections of slow theta, fast theta, and gamma bands during item retrieval. (*A*) Results for left hemisphere. Upper figure shows the comparison between slow and fast theta band. The connectivity of slow theta is compared with that of fast theta in each region during retrieval. Synchrous connectivity is shown in hot colors (red, orange), and desynchrous connectivity in cold colors (blue, light blue). Only the connections are shown when *P*-value is less than 0.05 with paired *t*-test. In synchrous connectivity, slow theta is represented with red, and fast theta shown with orange. In desynchrous connectivity, slow theta is shown blue, and fast theta represented with light blue. In the lower figure, red color indicates synchronous connections (connectivity preceding successful retrieval increased) and blue color shows desynchronous connections. (*B*) Results for right hemisphere.

**Figure 7:**
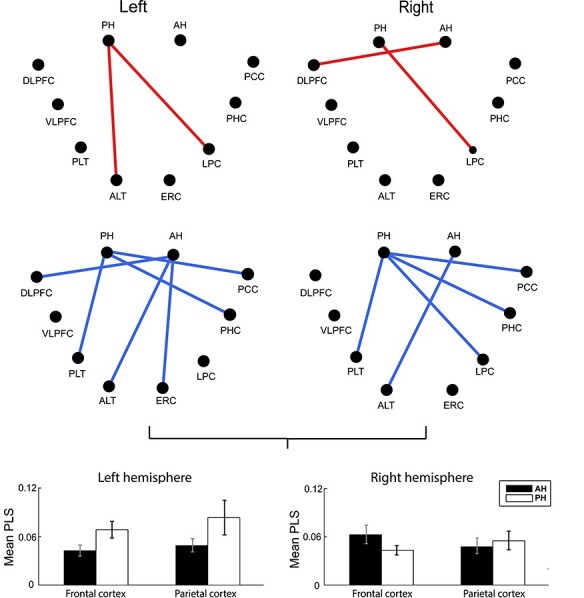
Direct comparison of functional connections for anterior versus posterior hippocampus during item retrieval. Red color of each edge represents a significantly greater synchronous connection and blue color indicates desynchrony. In this figure, only connections which are greater by direct comparison of z-scores for the anterior versus posterior hippocampus are plotted. The figure below shows the mean value of anterior and posterior hippocampus connections for frontal (dorsolateral prefrontal cortex and VLPFC) and parietal cortex (lateral parietal cortex and posterior cingulate cortex) analogous to plot for encoding. There is no significant difference between the anterior and posterior hippocampus both for either the frontal or parietal cortex (paired *t*-test, FDR corrected *P* > 0.05).

**Figure 8:**
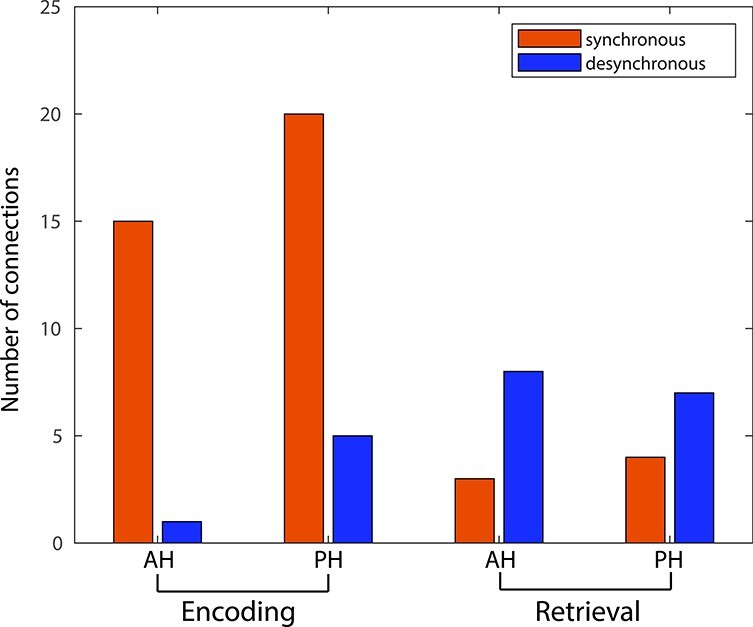
Number of synchronous and desynchronous connections in encoding versus retrieval. Number of significant synchronous functional connections (greater connectivity for successful trials) and significant desynchronous functional connections (decrease connectivity for successful trials) is summarized for encoding and retrieval.

### Memory Retrieval is Characterized by Sparse Synchronous Connections for Both Anterior and PH

We followed the same general approach when analyzing functional connectivity during item retrieval, although in this case the comparison was not between successful and unsuccessful study events but rather between the period of time immediately preceding successful recall and a baseline period during a “deliberation” phase of the task, temporally distinct from recall events following previously published methods (Long 2017). Connectivity across all frequency bands was generally decreased during memory retrieval compared to what we observed during encoding, with significant connections driven mainly by desynchronization effects (14 significant desynchronous connections versus 6 synchronous ones, Fig. 5). We summarized these effects in the same way, averaging the absolute value of functional connectivity weight (PLS) across all possible connections and all three frequency bands for anterior and posterior location; this is visible in Fig. 5. Even in the setting of a general decrease in connectivity, we observed that both the anterior and PH exhibited a connectivity increase with the LPC during memory retrieval, consistent with expectations regarding the role of this region in memory retrieval (Rugg and King 2018). However, the PH-frontal connectivity observed during item encoding was not present. Similarly, the pattern in the AH was reversed in terms of its connectivity with the PCC (exhibiting decreased connectivity during retrieval compared with increased connectivity during encoding).

### Fast Theta Desynchronization Characterizes Hippocampal Networks During Memory Retrieval

Fig. 7 shows those connections for which the connectivity weights were significantly different when directly compared between anterior and PH (pairwise by subject), as with encoding. AH/PH connectivity with the LPC is significantly stronger for the PH in the slow theta band (although both the anterior and PH exhibited significant connectivity [as in Fig. 6], this was significantly greater for the PH). Interestingly, PH–LPC connectivity was significantly more desynchronous in the fast theta band, but more synchronous in the slow theta and gamma bands. We discuss below the relevance of this finding to the model of theta desynchronization developed from previous investigations of connectivity during episodic memory processing. We repeated our comparison of parietal versus frontal connectivity measurements using retrieval data. Results are shown in Fig. 7. As suggested by the connectivity differences, results during retrieval did not reveal a significant A/P hippocampal/cortical interaction (*F*(1,48) = 1.17, *P* > 0.05, η^2^ = 0.02), unlike during encoding. During memory retrieval, we observed a significant effect of frequency band on connectivity for both the anterior and PH (main effect of frequency band, *F*(2,95) = 10.03, *P* < 0.05, η^2^ = 0.17). This pattern is visible in the pairwise connectivity comparisons (between slow and fast-theta oscillations for significant connections, as described above for encoding), as shown in Fig. 6. Synchronization effects in both the anterior and PH were stronger for both frontal and parietal locations in the slow theta band, while desynchronization occurred in the fast-theta range especially in the left hemisphere.

### Distinct Functional Connectivity Patterns Support Encoding Versus Retrieval

Based in part on observations of unique retrieval-related networks in episodic memory paradigms, we hypothesized that we might observe significant differences in encoding versus retrieval hippocampal networks. In aggregate, the proportion of significant synchronous connections across all frequency bands was greater for both the anterior and PH during encoding but we observed relatively more desynchronous connections during memory retrieval (Fig. 8). We directly compared the ratio of synchronous versus desynchronous connections between encoding and retrieval and found that this difference was significant (Chi = 12.08, *P* < 0.05). Although substantial caveats must be held in mind when comparing encoding versus retrieval patterns in free recall (addressed below in the Discussion section), this stark contrast in patterns suggests that encoding and retrieval networks differ substantially.

## Discussion

In this analysis, we directly address two key uncertainties of human hippocampal functioning using direct brain recordings: what differences in functional connectivity exist between the anterior and PH in episodic memory, and how do these differences interact with connections in different theta frequency ranges? Our key findings carry implications in three domains: 1) anatomical models of mnemonic processing; 2) the role of theta oscillations during encoding and retrieval; and 3) models of hippocampal longitudinal specialization.

### Implications for Models of Mnemonic Processing

Our first key finding related to mnemonic processing is that distinct anterior versus posterior hippocampal networks support memory encoding. We observed this in two respects. First, in the right hemisphere, aggregate posterior hippocampal connectivity is significantly greater than anterior connectivity (Fig. 2). Second, in the left hemisphere, encoding is characterized by posterior hippocampal–prefrontal connections (to DLPFC and VLPFC) and anterior hippocampal–parietal connections (to PCC and LPC). This was surprising in part because it seems to run counter to predictions of the AT/PM system hypothesis, one of the principal theoretical treatments of human memory that motivated our analysis (Ritchey et al. 2015). In brief, this model proposes that the PM system supports spatial navigation, construction of “schema” integrating contextual and item level information and overlaps with default mode regions potentially favoring activation during memory retrieval. By contrast, the AT system is more heavily implicated in semantic processing, and the human data using the free recall paradigm suggests semantic clustering (as compared to temporal clustering) favors activation within the AT system (Kragel et al. 2019). The original theoretical treatment of the model does not assign anterior or PH to one or other of the systems, but a priori, we had expected to observe stronger AH–prefrontal connectivity based upon the presence of the uncinate and occipitofrontal fasiculi to connect these areas. However, presumed posterior–prefrontal connectivity mediated by PHC and medial entorhinal regions has been connected to memory processing in rodents (Preston and Eichenbaum 2013), consistent with our findings. Taken together, these observations specify how the AT/PM model may map onto hippocampal–cortical interactions and potentially support the differentiation of left versus right hemisphere connectivity predictions in subsequent revisions (at least for verbal stimuli).

Our observations of hippocampal connectivity during memory retrieval more closely align with predictions of the AT/PM model, especially the observation that both the anterior and PH exhibit significant connectivity with the LPC (including the angular gyrus, a key participant in the proposed PM system) (Fig. 5). This occurred specifically in the left hemisphere. Noninvasive imaging studies indicating a key role for the left angular gyrus in memory retrieval predict the existence of such a connection (Seghier 2013), and the fact that the connectivity is found for both anterior and posterior hippocampal regions is an important finding in our data (although these connections were stronger for the PH, Fig. 7). Additionally, we did not observe strong differences in parahippocampal connectivity differences between anterior versus posterior regions, which also accords with participation of the hippocampus within both the AT and PM systems (Ward et al. 2014). Entorhinal connectivity was overall weak in the present analysis, which may suggest that such connections are not strongly modulated by encoding success or that a more fine-grained quantification of connectivity is necessary (perhaps one that does not collapse across all possible electrode–electrode pairs for a given subject), or potentially with more detailed anatomical segregation of entorhinal regions, as has been proposed (Baird et al. 2013).

### Implications for the Role of Theta Oscillations in Mnemonic Processing

The study of theta oscillations is critical because models of episodic memory derived from rodents rely on theta to provide a signal that coordinates local activation across the cortex in a way that may be propitious for LTP formation, memory consolidation, and recall (Uzakov et al. 2005). Existing human observations have provided equivocal information regarding the importance of theta oscillations and their preferred frequency. While numerous episodic memory and spatial navigation studies have suggested that oscillations in the 2–5 Hz range are important for human memory (Klimesch 2018; Jacobs 2014), other studies have reported oscillations in faster theta ranges, especially involving recognition memory (Nyhus and Curran 2010). In rodents, there is evidence for two distinct types of theta oscillations, namely type I theta which appears during movement, sniffing, or other kinds of environmental exploration and is generally considered the most memory sensitive, and type II theta that appears during motionless anxiety and is thought to be less related to memory formation (Bland 1986). Confusingly, both 2–5 and 4–9 Hz theta oscillations have demonstrated such properties across various investigations in different memory paradigms (Roberts et al. 2013; Zhang and Jacobs 2015).

A critical feature of our analysis was the separate examination of slow versus fast theta oscillations. We first establish that both fast (5–9 Hz) and slow (2–5 Hz) theta oscillations are present in the anterior and PH (consistent with previous data [Lega et al. 2011; Watrous et al. 2011]), and further that these oscillations are present simultaneously within individual subjects. This complements previous findings and in our estimation reinforces the need to consider these oscillations as distinct entities and test for separate functional properties when using hippocampal recordings.

In our analysis, evident fast versus slow theta differences occurred during memory retrieval, in which sparse connectivity increases occurred in the slow theta/gamma range while fast theta oscillations exhibited general desynchronization across several regions. During encoding, however, we observed similar aggregate connectivity across all bands. This aggregate finding, however, hides the fact that individual connections may be stronger for fast versus slow theta oscillations. Fig.s 3 and 6 show connections that are significantly different between fast and slow theta oscillations, indicating that for specific connections, one or the other band may be more mnemonically relevant. Our findings also inform the understanding of theta/gamma connectivity differences. Previous studies examining brain-wide connections during episodic memory encoding identified an overall theta synchronization/gamma desynchronization pattern (Solomon et al. 2017). Hippocampal connections formed a small subset, and did not differentiate anterior versus PH. While we did observe such patterns for some connections (for example, lateral temporal cortex, see Fig. 2) during encoding, synchronous gamma connections predominated overall. During retrieval, the key hippocampal–lateral parietal connection is synchronous for both slow theta and gamma connectivity (desynchronization occurs in the fast theta range). Observed differences in hippocampal versus cortical activity during memory processing along with the connectivity results discussed above motivated an influential theory, termed “complementary learning systems” or CLS, originally formulated by McNaughton and subsequently elaborated using more recent intracranial data (Hanslmayr et al. 2016; McClelland et al. 1995). The recent formulation of the model incorporates differences in oscillations in the hippocampus, in which theta and gamma synchronization occur, versus the neocortex in which local desynchronization in lower frequencies supports memory processing. Our findings complement this model and indicate which specific connections mediate cortical–hippocampal interactions. We elected to focus our results on hippocmapal connectivity patterns, but a more comprehensive picture of cortical–hippocampal interactions would ideally incorporate a multivariate approach including local power changes and connectivity results when characterizing encoding and retrieval networks. However, previous investigations have not demonstrated consistent correlation between PLV-based connectivity measurements and oscillatory power effects (Solomon et al. 2017). A specific prediction of the updated version of the CLS model described by Hanslmayr et al. (incorporating oscillatory data) is that brain regions exhibiting local desynchronization (manifest as power differences) would demonstrate increased connectivity with the hippocampus. Our findings entail that such complex analyses must take account of longitudinal and theta frequency differences.

### Implications for Models of Hippocampal Longitudinal Specialization

In a parallel line of investigation, researchers have observed consistent differences in relative contributions of anterior versus posterior hippocampal activity toward different behaviors. In humans, this has variously been proposed to include differences according to emotional task requirements (Zeidman and Maguire 2016), spatial versus nonspatial reasoning (Epstein et al. 2017), encoding versus retrieval activation (Greicius et al. 2003), and “gist” versus detail level mnemonic processing (textcolorblue Moscovitch et al. 2016; Poppenk et al. 2013). Our own observations in humans performing an episodic memory task suggest that while gamma oscillations exhibit task-related modulation across the hippocampal axis, slow theta oscillations may be more mnemonically sensitive in the PH (Lin et al. 2017).

We did not observe a pattern consistent with distinct frequency specific connectivity preference organized along the longitudinal axis. In other words, we did not observe that the PH consistently exhibits connectivity in slow theta, while AH exhibits fast theta connectivity, or vice versa, for example. Unexpectedly, we observed significantly greater posterior hippocampal connectivity in the right hemisphere. One possible explanation is that left hemisphere regions exhibit more subtle connectivity relationships involved in representing specific item details, with a right hemispheric contribution that is more similar across item types and therefore more prominent in our analysis. Observations of posterior hippocampal activity that predicts spatial navigation success may support this view (Herweg and Kahana 2018), although this is admittedly speculative and will require anterior/posterior connectivity analyses that disambiguate semantic versus temporally mediated encoding mechanisms or other memory types.

While we observed stronger posterior hippocampal connectivity during encoding, we did not find evidence to support “exclusive” encoding-related connectivity in the AH but retrieval-related connectivity in the PH (see above related to hippocampal–parietal connectivity). Such an observation would have been consistent with previous proposed anterior/posterior hippocampal differences. Likewise, the significant posterior hippocampal connections, we observed in this verbal memory task, suggest that models positing a spatial/nonspatial distinction for anterior/posterior differentiation are at best incomplete (Poppenk et al. 2013). Our data cannot directly test the gist/detail model proposed by Poppenk, as free recall does not control the granularity of individual memory items. However, the finding that anterior/posterior hippocampal connectitivity differences occur in a task without gist versus detail-type differences will need to be accounted for in updated versions of the granularity model. The use of task-based functional connectivity measures should perhaps be emphasized in this regard.

We acknowledge several important caveats when interpreting our results. The first is that the connections we report represent those that are modulated by encoding and retrieval success, which implies that important connections may exist (for example between the AH and the entorhinal region) that are not strongly modulated by memory performance. For the same reason, one must keep in mind that in free recall encoding-related success effects may fundamentally differ from retrieval-related success effects. However, previous investigations have demonstrated recapitulation of oscillatory patterns between encoding and retrieval consistent with reinstatement of item-related activity at the time of recollection (Manning et al. 2011). In our data, we observed synchronous connectivity between the hippocampus and the lateral temporal cortex during both encoding and retrieval, which is consistent with previous experiments identifying reinstatement effects in the temporal lobe. Overall differences in connectivity patterns and the synchronous hippocampal-lateral parietal connection, we observed lend support to noninvasive studies positing a core episodic retrieval network, different than what one might hypothesize using a more general model of memory reinstatement. We also wish to emphasize that further investigation is necessary to establish differences in theta connectivity versus relevant functional connections for the hippocampus in other subgamma frequency ranges, especially alpha and beta bands (Herweg et al. 2016; Hanslmayr et al. 2016).

Further, we aggregated signal across multiple electrode pairs in order to use a single connectivity metric per subject per region pair, and aggregated data across all subjects for overall anterior/posterior comparisons. We believe that this approach permits generalizability of our findings and reduces the impact of heterogeneity in our data but at the expense of diluting more subtle or specific region–region connections. This aggregation also required us to collapse signal from relatively broad cortical areas such as the VLPFC that may mask more specific connections visible using more fine-grained anatomical segregation. However, the benefit of this aggregation was the ability to perform a pairwise anterior/posterior comparison, which we believe adds significant impact to our findings. Another potential concern is the possible effect of the (necessary) use of epilepsy patients in our analysis. While none of the subjects had radiographic evidence of mesial temporal sclerosis, we explicitly excluded any subjects who did not have both anterior and posterior hippocampal electrodes out of concern for the influence of differences in sampling across different patients affecting observations. Insofar as any region included in our analysis exhibited epileptic properties, these areas were interacting with both the anterior and PH. Certainly, other studies using human participants in the same fashion suffer from similar limitations, and the need to address critical questions of hippocampal longitudinal specialization makes our findings valuable in spite of these concerns. Our findings may ultimately impact the clinical care of patients with temporal lobe epilepsy insofar as accurate models of longitudinal specialization may help predict the impact of temporal lobe surgery. In the current clinical practice, a common question is whether to pursue standard temporal lobe resection versus laser-assisted amygdalohippocampectomy. The latter often entails greater posterior hippocampal residual tissue which may ultimately be favorable if posterior hippocampal functioning is relatively preserved prior to surgical intervention.
